# Red flags for thoracic endometriosis

**DOI:** 10.36416/1806-3756/e20250229

**Published:** 2025-09-22

**Authors:** Lorenzo Carriera, Roberto Lipsi, Angelo Coppola

**Affiliations:** 1. Catholic University of Sacred Heart, Pulmonology Department, Rome, Italy.; 2. Department of Pulmonology and Sub-Intensive Respiratory Unit, Ospedale Santa Maria della Misericordia, Perugia, Italy.; 3. Department of Pulmonology and Sub-Intensive Respiratory Unit, Ospedale San Filippo Neri-ASL Roma 1, Rome, Italy.

**Figure 1:**
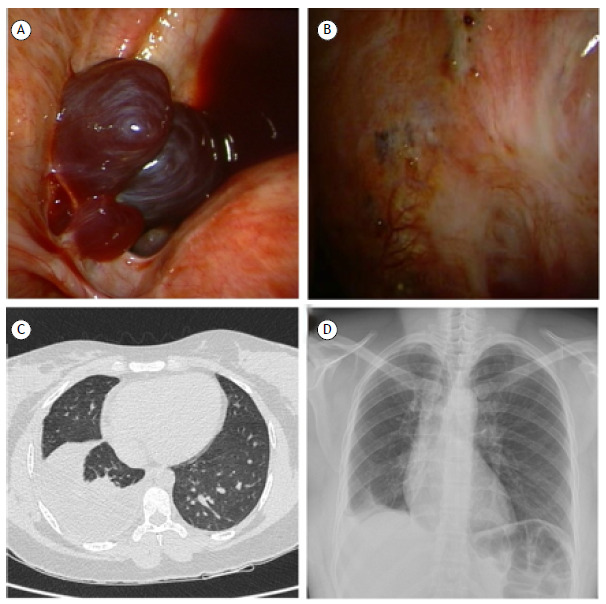
A) Wine-red nodules on the diaphragm visualized during medical thoracoscopy; B) Small punctiform wine-red lesions on the parietal pleura; C) Chest CT at admission, showing right-sided basal pleural effusion; D) Chest X-ray 1 month after the initiation of gonadotropin-releasing hormone therapy.

A 38-year-old Brazilian woman with a previous diagnosis of pelvic inflammatory disease presented to our hospital with a two-month history of dyspnea, chest pain, and dry cough. Chest CT revealed right-sided basal pleural effusion. The QuantiFERON-TB test result was positive. On the basis of those findings and the symptoms, we suspected a diagnosis of pleural tuberculosis. An explorative thoracentesis was performed. The aspirated pleural fluid was hemorrhagic. The cytological examination showed a predominance of histiocytes and leukocytes, and no malignant cells were detected. At that point, we decided to perform medical thoracoscopy to obtain biopsy specimens from the parietal pleura. The procedure revealed thickened pleura and small, wine-red lesions on the diaphragm and parietal pleura (Figure 1), findings suggestive of pleural endometriosis. Histopathological analysis confirmed the presence of epithelial glandular elements, and immunohistochemical staining was positive for CD10 and estrogen receptors. Thoracic endometriosis presents nonspecific symptoms, making the diagnosis challenging. In women of reproductive age presenting with chest pain and pleural effusion, endometriosis should be considered as a possible cause.^1^ Hormone suppression therapy with gonadotropin-releasing hormone analogs can help alleviate symptoms as well as improving the overall quality of life and daily functioning.^2^ Early recognition and multidisciplinary care are key to achieving better outcomes.^3^
